# Dimeric interactions and complex formation using direct coevolutionary couplings

**DOI:** 10.1038/srep13652

**Published:** 2015-09-04

**Authors:** Ricardo N. dos Santos, Faruck Morcos, Biman Jana, Adriano D. Andricopulo, José N. Onuchic

**Affiliations:** 1 Center for Theoretical Biological Physics, Rice University, Houston, TX 77005-1827; 2 Laboratório de Química Medicinal e Computacional, Instituto de Física de São Carlos, Universidade de São Paulo, São Paulo, São Carlos, 13563-120, Brazil; 3 Department of Physical Chemistry, Indian Association for the Cultivation of Science, Jadavpur, Kolkata-700032, India

## Abstract

We develop a procedure to characterize the association of protein structures into homodimers using coevolutionary couplings extracted from Direct Coupling Analysis (DCA) in combination with Structure Based Models (SBM). Identification of dimerization contacts using DCA is more challenging than intradomain contacts since direct couplings are mixed with monomeric contacts. Therefore a systematic way to extract dimerization signals has been elusive. We provide evidence that the prediction of homodimeric complexes is possible with high accuracy for all the cases we studied which have rich sequence information. For the most accurate conformations of the structurally diverse dimeric complexes studied the mean and interfacial RMSDs are 1.95Å and 1.44Å, respectively. This methodology is also able to identify distinct dimerization conformations as for the case of the family of response regulators, which dimerize upon activation. The identification of dimeric complexes can provide interesting molecular insights in the construction of large oligomeric complexes and be useful in the study of aggregation related diseases like Alzheimer’s or Parkinson’s.

The ability of life’s basic components to act synergistically, make decisions and perform complex functions is essential in biology. For the case of proteins, these interactions have been shaped by evolutionary pressures constrained by their three dimensional structures and their physiological requirements. A considerable fraction of biological processes in cells are performed by protein complexes that result from stable interactions between subunits of equal or different compositions. Important examples include scaffolding proteins like actin or tubulin forming actin filaments and microtubules^1^,^2^,^3^,^4^, macrocomplexes forming rings used in cell division (tubulin-like FtsZ protein)^5^, protein degradation (FtsH AAA protease)^6^,^7^ and dimerization occurring in transcription factors required to bind DNA and perform gene regulation^8^,^9^. All of these protein complexes are examples of the prevalent group called homo-oligomers^10^. Furthermore, homodimeric systems are the most abundant inside this subgroup^10^,^11^ and seem to have, in average, twice as many interaction partners than non-self-interacting proteins^12^. Dimeric interactions are ubiquitous as well as relevant for cell survival, yet there is no indication in their amino acid sequences whether a given sequence will or will not form a dimer. Even when experimental evidence points towards dimer formation, it is a challenge to determine the molecular details of the complex. Dimeric interactions have to satisfy both monomeric and dimeric structural requirements and we show that these constrains are reflected as direct amino acid couplings in the collection of sequences of a given protein family. The idea of coevolution has been utilized to study residue-residue covariation and its implications in residue pair energetics^13^,^14^,^15^,^16^,^17^,^18^. This idea has been useful to predict the structure of protein monomers^19^,^20^,^21^,^22^,^23^, especially in combination with knowledge potentials used to parameterize inter-residue distances^24^,^25^, and to uncover conformational plasticity^26^,^27^. Residue coevolution has also been studied in the context of protein-protein interactions. An early application was our prediction of complexes between histidine kinases and response regulators^28^,^29^. Other studies include specificity in two component systems^30^,^31^, the prediction of interaction modes for novel cancer targets^32^, macro-complex formation and function of the AAA protease^33^ and several other diverse complexes^34^. Most of the applications of coevolutionary methods to study heterodimeric interactions involve the construction of a database of known heterodimer pairs using a priori knowledge. For instance, the assumption that two protein sequences that are proximally encoded in the genome interact or the presence of single copies in the organism facilitates this matching. Homodimers are a special case where the interacting signal is obtained from sequences belonging to a single family. The challenge is that coevolving residues with high couplings reflect mostly physical contacts of the monomeric three dimensional structure. Hence, dimeric or oligomeric contacts are mixed with monomeric contacts making it challenging to distinguish between them.

Prediction of dimeric complexes is an area of active research with progress done from different fields including docking algorithms^35^,^36^,^37^,^38^,^39^,^40^ as well as molecular dynamics simulations^41^,^42^,^43^,^44^. Here, we show that a relatively simple protocol can be used to extract important coevolving dimeric contacts from the monomeric signals obtained using Direct Coupling Analysis (DCA)^22^ and that those couplings can be used to predict complexes with high accuracy. Although this concept might not be applicable to all proteins, here we provide evidence that this approach works for a set of 18 different dimeric complexes from different families which cover different classes, folds, conformations as well as complexes with multidomain architectures with different sizes including medium to large proteins (up to 446 aa). Our success for this diverse set of families and classes suggests that this methodology can be applied to many other protein systems with rich sequence data, without the requirement of genome adjacency or single copies in the organism. Although not universal, the applicability of this idea to a larger number of molecular systems where dimerization or oligomerization plays an important biological role, but the molecular details have not been elucidated yet, is possible as the number of available sequence information increases over time.

## Results

**Figure 1:**
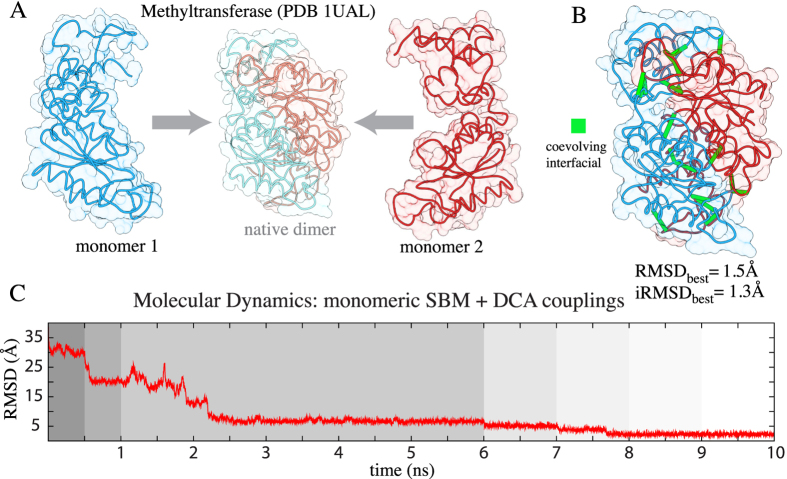
Inferring dimerization complexes with coevolutionary pairings. (**A**) Two monomeric structures of the tRNA methyltransferase are used in a molecular dynamics simulation that brings the molecules together until reaching a stable complex close to the native homodimer state (shown in the center with light colors). (**B**) Accurate complex formation is driven by the dimeric constraints (shown in green) extracted using DCA. This methodology seems robust to the existence of those non-dimeric contacts that are used as constrains from DCA. (**C**) The RMSD progression of the simulation shows how at different stages of the protocol (shown in different background colors) the procedure gets closer to the native structure. At each stage the equilibrium distance and the shape of the Gaussian function are parameterized (See Supplementary Methods) to facilitate the satisfiability of the DCA couplings. For example, the contact range starts at 50 Å and concludes at typical native distances of 8 Å. This figure is representative of all the systems investigated here. For other RMSD progression plots refer to Supplementary Fig. S1.

The key idea to study residue-residue coevolution involved in dimerization is the combination of accurate prediction of residue contacts using DCA with the availability of monomeric structural data (e.g. X-ray crystallography or NMR). This provides a natural filter for residue pairs that are highly coupled but are found in the hydrophobic core of the protein. These direct couplings are most probably pairings required for folding and not for complex formation. Therefore, we exclude highly coupled pairs that have low surface accessibility as well as those pairs that are in close proximity in the monomeric contact map. Although there exist dimeric contacts that are both monomeric and dimeric, filtering them appears to have a small effect on the complex prediction accuracy. The resulting contacts are then incorporated in a coarse-grained (Cα) SBM with Gaussian potentials^45^ for complex formation. Figure 1 shows a summary of this methodology exemplified by the tRNA methyltransferase dimer. The residue-residue contacts obtained from coevolution bring the two molecules together after an annealing-like procedure needed for a controlled interfacial reordering and binding. Figure 1B shows the most accurate predicted complex with a lowest RMSD value of 1.5 Å and Fig. 1C shows the RMSD progression until reaching a stable complex close to the native state. The details on how to extract coevolving dimeric signatures and a description of the parameters used in the binding simulations are described in the *Methods* section and Supplementary Methods.

### A case study of dimeric protein complexes in several protein families

We first studied 16 dimeric systems for which we have known complex structures needed for model validation. We selected proteins that are typically larger than proteins used for folding simulations, ranging from 121 to 444 aa with an average of 303 aa. These proteins belong to families with abundant number of sequences (>2500) and very distinct folds and structures. Table 1 lists the dimeric proteins used in this study along with their characteristics and their respective protein families.

**Figure 2:**
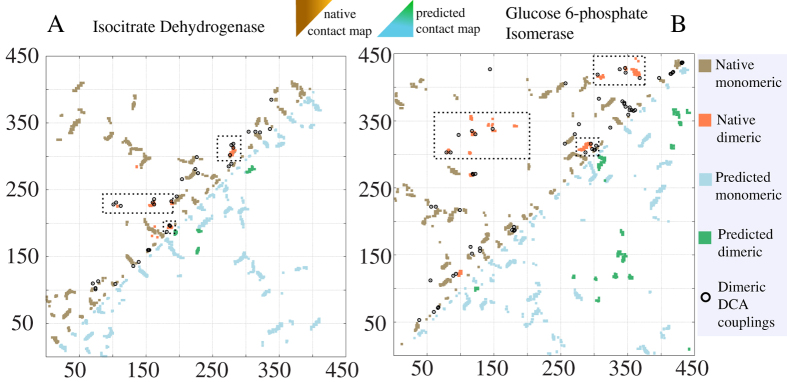
Direct couplings of single domain families contain signals of dimeric interfaces. (**A**) Contact maps of protein isocitrate dehydrogenase (412 aa). The upper triangular map shows the native monomeric contacts (brown) along with the native dimeric contacts (orange). The circular symbols represent the top ranked couplings estimated using DCA, the solvent accessibility criterion and removing contacts close to the monomeric map. These DCA couplings are used as constrains in the molecular dynamics simulation. The lower triangular map shows the contacts of the best predicted complex obtained after using the SBM + DCA protocol. Monomeric contacts are shown in blue and resulting dimeric contacts in green. (**B**) Contact maps of glucose 6-phosphate isomerase use the same convention as in (**A**) with similar and consistent results. The predicted contact maps for the protein-protein interface colored in green are very similar to their native counter parts shown in dashed boxes.

**Figure 3:**
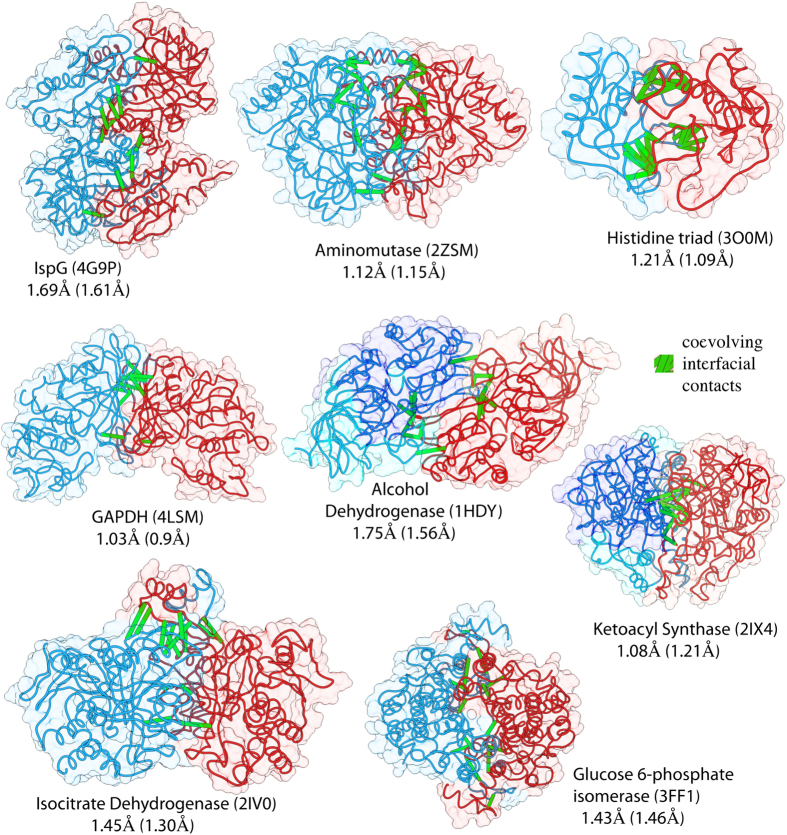
Predicted dimeric structures for 8 different proteins and families. The best inferred bound complexes have different topologies and sizes. These proteins have lengths ranging from 121–444 aa (mean 303 aa) and contain distinct folds as well as single and multidomain architectures (ketoacyl synthase and alcohol dehydrogenase). A notable case is the protein GAPDH for which the iRMSD has sub-angstrom resolution and the ketoacyl synthase with an RMSD = 1 Å. For the case of the isocitrate dehydrogenase we see that the dimeric interface shown on the top requires a conformational rearrangement in order for the helices to wrap around each other. This was only possible given the high number of coevolved contacts found around this area. See Supplementary Fig. S4 for more systems.

Figure 2 depicts native contact maps of two different dimeric complexes: protein isocitrate dehydrogenase (2IV0) and glucose 6-phosphate isomerase (3FF1) along with the contact map of the predicted dimer structure. The native maps, shown in the upper triangular section of the plot, have two different types of contacts. Native monomeric contacts are colored in brown and native multimeric contacts are colored in orange. These maps also show the predicted dimeric contacts from DCA in black circles. The exact number of constrains used for each case depends on filtering the top 100 DCA pairs using a solvent accessibility criterion and by removing contacts around the monomeric structure (see *Methods* section). It has been suggested that these types of long distance couplings might be related to elastic interactions^46^ but this remains to be characterized. The number of couplings used in the simulations range from 30–75 for all the systems. Most of the remaining predicted contacts are part of the dimeric interface and are used as contact pairs described by a Gaussian potential (Supplementary Methods, Eqs 6–7). The lower triangular regions on the contact maps of Fig. 2 represent the contacts of the best-predicted complexes. The intra-domain contacts are shown in blue and the intermolecular contacts are shown in green. The reconstruction of these maps is highly accurate and recapitulates well both intra and inter-domain native interactions. The contact maps for the remaining proteins studied are shown in Supplementary Figs S2-3 and the estimated complexes are shown in Fig. 3 and Supplementary Fig. S4.

Using the same protocol as shown in Fig. 1, we estimated dimeric complexes for the remaining 13 systems. For all the different experimental dimers studied our predictions had an average root mean square deviation for the best complexes of RMSD_best_ = 1.60 Å (mean interfacial iRMSD_best_ = 1.29 Å). If we consider the average RMSD at the last stage of the annealing procedure (see Fig. 1C) when the distance parameter for contacts is 8 Å, the mean RMSD_r=8Å_ = 2.80 Å (mean iRMSD = 2.21 Å). See Supplementary Table S1 for individual values. Figure 3 shows the structures of the predicted complexes for proteins IspG (4-hydroxy-3-methylbut-2-en-1-yl diphosphate synthase), aminomutase (glutamate-1-semialdehyde aminotransferase), histidine triad, tRNA methyltransferase, GAPDH (glyceraldehyde 3-phosphate dehydrogenase), alcohol dehydrogenase, glucose 6-phosphate isomerase and ketoacyl synthase. The monomers are colored in blue and red and the true positive dimeric contacts driving the complex formation are shown in green. RMSD and iRMSD (in parenthesis) are shown as performance metrics (see also Supplementary Fig. S4 for the predictions of further protein complex structures). For proteins alcohol dehydrogenase and ketoacyl synthase there is a multidomain architecture, hence in addition to estimate dimeric contacts as described before we also estimated contacts across different domains. Since these domains belong to the same protein, the sequence pairing procedure is trivial and the results are equivalent as cases of heterodimers^29^,^31^,^34^.

### Prediction of multiple dimeric complexes in response regulator proteins

**Figure 4:**
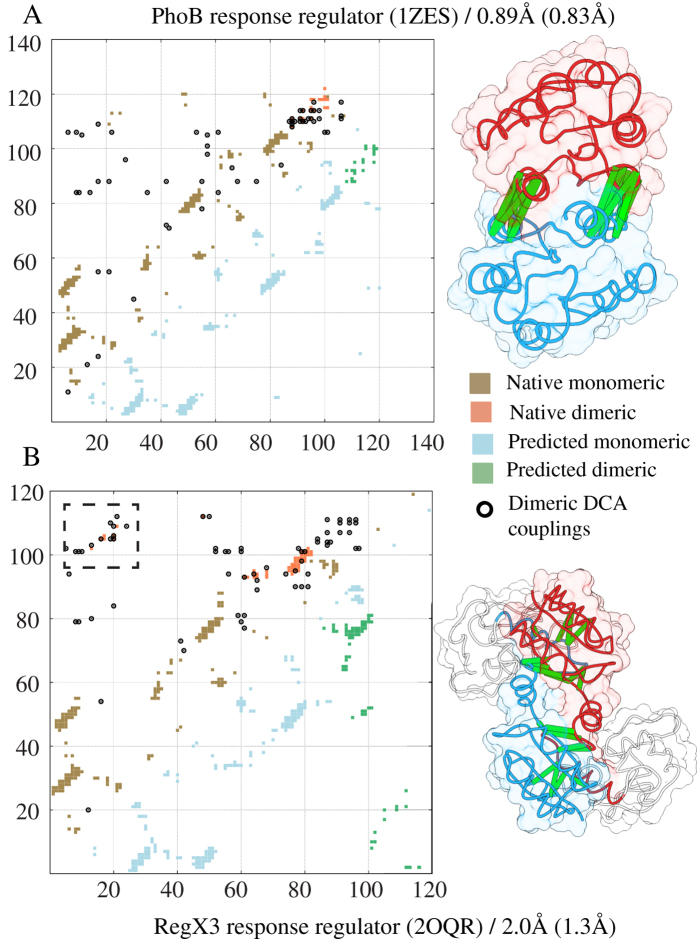
DCA/SBM for interfaces can infer multiple dimer conformations. (**A**) Coevolving contacts for the active state of the PhoB response regulator dimer in *E. coli* shown in black circles overlap well with the dimeric interface (orange). The predicted complex has a resolution of RMSD = 0.89 Å (iRMSD = 0.83 Å). (**B**) An alternative configuration of the activated dimer in protein regx3 upon phosphorylation is also predicted with an RMSD = 2 Å (iRMSD = 1.3 Å). This configuration involves domain swapping of helix α4 and α5 and sheet β5 and a second interacting region (dashed box) that is also captured by DCA. This structure also includes the effector domain (shown in white) that is a member of the transcriptional regulatory protein, Trans_reg_C (PF00486). Domain swapping is possible because of the contacts formed between the effector domain and the receiver domain in regx3 that allow this helical region to be exposed for binding.

Response regulators are members of a very large family of primarily prokaryotic proteins with more than a hundred thousand members. They are involved in signaling pathways where their receiver domain (Pfam PF00072) is typically phosphorylated by a histidine kinase (Pfam PF00512). This event triggers a conformational change and promotes dimerization of the phosphorylated protein, activating its function as a transcription factor that binds to DNA and continues a cascade of events in response to its original input sensed by the kinase^47^,^48^,^49^. Homodimerization of the receiver domain (REC) is fundamental to achieve an active state conformation^50^. We studied the phosphate regulon transcriptional regulatory protein PhoB in *E. coli,* which upon activation dimerizes in its typical configuration (α4-β5-α5). Figure 4A shows the result of applying the SBM + DCA methodology to the complex formed by the REC domain of PhoB upon activation. A series of dimeric contacts among residues in the region 90–120 (orange) are detected by DCA (black circles). The complex was predicted with an RMSD accuracy of 0.89 Å with respect to the crystal structure (PDB 1ZES) for the best case and an average of 1.57 Å for the last stabilized simulation stage. This suggests the presence of a clear coevolutionary signal for the active state complex formation. It has also been suggested that the active state dimers for the REC domain of the response regulator can take alternative conformations. One of such conformations involves domain swapping of helices α4 and α5 and sheet β5, as well as the formation of distinct dimeric contacts^51^. We applied our methodology to the monomeric structures of the sensory transduction protein regx3 of *M. bovis* (PDB 2OQR) that binds using this alternative active interface. The contact map in Fig. 4B shows that some of the monomeric contacts in Fig. 4A become dimeric for regx3 and are highly coupled. Additionally, another region of contacts involving residues 10–20 interacting with residues 100–110 is also captured using coevolutionary analysis (see Fig. 4B, dashed box). These two contact regions drive the formation of this alternative complex with a resolution of RMSD of 2 Å (iRMSD = 1.33 Å). Regx3 is a multidomain protein containing an effector domain. If we compute the RMSD only for the response regulator domain, as in the case of PhoB, then the best RMSD = 1.13 Å. This implies that coevolutionary signals for multiple dimeric conformations are present and can be used to characterize multiple physiologically relevant configurations. Although the dimeric state of the receiver domain is mainly observed for the activated state, some studies suggest that an inactive state can also form homodimers and some symmetric units supporting this view^50^,^52^. Nonetheless, it is not known if these inactive homodimers are formed *in vivo* or if they have any physiological relevance. Furthermore these inactive state complexes are arranged in such a way that the aspartate residue that is phosphorylated upon activation is not accessible to the kinase making this configuration less physiologically viable. Although some of the contacts in the inactive state dimer are captured by DCA, they do not appear to be sufficient to reach the same resolutions as for the active states. Supplementary Fig. S5 shows the predicted structure for such system, which has no resemblance to the inactive state dimeric interface observed in PDB 1B00. One interpretation of this result is that our methodology is not able to capture this alternative dimer correctly. The other view is that the evolutionary signal for this dimeric inactive state is weak and therefore not functional.

Most of the protein complexes presented here were validated with experimental structures that were already part of a dimer. To understand the effects of estimating complexes using only monomeric information that might be different from their bound structures, we have studied additional systems where the knowledge of a monomeric structure is available. We predicted complexes for the response regulator PhoB (monomeric PDB 1B00) and compared against the dimeric complex described before (PDB 1ZES); the Zucchini endoribonuclease (monomeric PDB 4GEN, dimeric PDB 4GEL) as well as the Inositol monophosphatase (monomeric PDB 2QFL and dimeric PDB 2HHM). We observed that, in average, the RMSD resolution is decreased by 1.2 Å and 0.86 Å at the interface (see Supplementary Table. S4 and Figure S6). These results gave us confidence of the applicability of our method to novel cases where the structural information is not complete.

## Discussion

The use of coevolutionary information to study protein structure and molecular interactions is promising and has been the topic of recent research. The number of coevolutionary constrains needed to accurately reconstitute complexes is much smaller compared to protein structure prediction, making it a particularly promising application to study intermolecular interactions. Homo-oligomerization is prevalent across the molecular biology of the cell and relevant for a wide range of molecular functions from scaffolding to gene regulation. The results discussed in this work provide support that a combination of structure based modeling in concert with coevolutionary signals let us uncover dimeric complexes close to experimental accuracy. Our previous work has focused on specific and biologically relevant heterodimers^29^,^31^,^32^. Here we systematically advance this idea to homodimers with diversity in families and folds (see Supplementary Table S5) that present the additional challenge of having compound evolutionary signals with couplings required for monomeric structure formation that could lead to non-physiological complexes. We found that such distinct dimeric signals can be successfully extracted and be used to study protein interactions.

In addition to the requirement of sequence availability, the success of our methodology can be limited by the requirement of “mirrored” residue interactions of the same amino acid positions in both monomeric chains that are not detectable by coevolutionary methods. In the systems presented here, we do encounter these kinds of interactions but they are often mixed with typical cross-residue interactions that are detected and successful complex formation was driven by those interactions. We also observe a change in performance when we use monomeric structures to predict already known dimeric complexes. This performance change is mainly due to conformational differences between the monomeric experimental structure and the dimers. This effect is relatively small especially at the interface and in some cases, like the Zucchini endoribonuclease, negligible. Domain swapping might also present a challenge since important monomeric intradomain interactions might be missing in this protocol, however, this could be alleviated by also incorporating intradomain couplings. This, nonetheless, is a topic of further research.

Our methodology also allows us to uncover multiple dimer conformations as for the case of the active response regulator dimer. This opens the possibility of exploring a larger spectrum of complexes. This knowledge could be of use in rational drug design when the objective is to disrupt alternative dimeric interfaces or devising important residues for dimerization. Uncovering functionally relevant dimeric interactions are of great importance for structural assemblies like microtubule formation or filament formation. Coevolutionary docking can be of use to accurately build very large assemblies that are hard to achieve using X-ray crystallography or NMR methods. Finally, homo-dimeric interactions are relevant for aggregation-induced ailments like Alzheimer’s, Parkinson’s or prion diseases^53^,^54^,^55^. Therefore, understanding the evolutionary nature of aggregation using protein energy landscape theory^56^ could help in the study of these degenerative disorders.

## Methods

### Sequence Alignments and Directly Coupled Residue Pairs

In order to predict coevolved dimeric contacts between monomers, the datasets of multiple sequence alignments for all families present within each selected protein were extracted from Pfam^57^. All Pfam datasets contain more than 3500 sequences (see Table 1), a prerequisite to ensure statistical significance and a substantial level of prediction accuracy from Direct Coupling Analysis (DCA). A list of the Pfam families used for DCA predictions and its respective proteins are shown in Table 1. To estimate directly coupled co-evolving residue-residue physical contacts we used the mean field implementation of DCA (mfDCA), as described by Morcos *et al.*^22^. In this method, individual sites are represented by frequencies and couplets in multiple sequence alignments are defined as single and pairwise probabilities. Further details of this formulation and its performance can be found in^13^,^22^. The ordered couplets based on the Direct Information (DI) value can be interpreted as a ranking of the plausibility that residues pairs are in contact in a three-dimensional protein structure^19^.

The positions of the Pfam alignments for each protein were determined using the *hmmscan* module from HMMER software, which employs Hidden Markov Models to perform alignments^58^. The DI ranked contacts from Pfam family sequences were mapped to their residues in the PDB protein structures using the output of *hmmscan* and an in-house mapping script. Due to the fact that the dimeric proteins predicted in these studies are homo-dimeric, the signals corresponding to the intermolecular dimeric interactions are mixed among the monomeric contacts intrinsic of each chain. In order to filter these monomeric DCA contacts and to obtain only the coevolutionary signals related to dimerization, we developed a filtering protocol comprised by two steps. First, for one of the homo-dimeric chains we calculated the Solvent Accessible Surface Area (SASA) of each residue using GetArea^59^. In general, a residue needs to be significantly exposed to the surface of a dimer in order participate in interactions to promote protein-protein association. We removed all predicted pairs that did not have at least one of their residues with a SASA larger than 50%, which is a minimum value for a residue to be classified as solvent accessible according to the GetArea server. In a second step, we selected the top 100 DI pairs after SASA filtering and removed all predicted contacts that occur in one of the monomers. This was performed by comparing the DCA contacts with the native contact map between C_α_ atoms of the native monomer structure at a cutoff of 8 Å. The remaining contacts were included as interaction forces in the topologies used for molecular dynamics simulations.

### Structural Modeling

All the homodimers used in this study were retrieved from Protein Data Bank (PDB)^60^. The PDB accession code for each structure is shown in Table 1. The resolution of the selected proteins varies from 1.55 Å to 2.50 Å, with an average resolution of 1.97 Å. Homodimers that present only a monomer in the crystallographic asymmetric unit were duplicated by rotation and translation operations to generate a symmetry mate corresponding to the missing monomer in the structure. These operations were performed using PyMOL 1.6 molecular graphics system. Missing loops were modeled using the SwissModel server having the same structure as a reference template^61^. Atoms that were not part of the protein chain were removed before employing these structures to SBM modeling simulations. In order to carry on binding simulations as an effort to retrieve the original structure, each prepared native homodimer was separated in two monomers by a distance of 50 Å and then randomly rotated 180 degrees in the axis between molecules to remove the initial native complex orientation. Other angles and axes of rotation were tried with no significant change in the performance of the protocol. The outcome structures were processed using the SMOG server to generate C_α_ models and structure-based (SB) potentials suitable to carry molecular modeling simulations with GROMACS 4.5.7 software^62^,^63^.

### Molecular Simulations

In order to reproduce dimerization of the selected complexes, the structure of each homodimer separated by 50 Å in two monomers was processed by the SMOG server, generating topologies containing SBM coarse-grained potentials. The DCA contacts obtained using the filtering process described before were utilized to generate structure-based models with Gaussian potentials describing residue pairs that should be in contact in the condition of minimum energy. For a detailed description of Gaussian potentials^45^ employed here see Supplementary Methods. The potentials were added into the topology generated by SMOG, alongside with the potentials related to the monomeric contacts, which were also parameterized in the Gaussian potential. To avoid substantial changes in each monomer conformation during the binding process, the dihedrals strength constant *k*_*d*_ was increased by a factor of 100 from its original value generated by SMOG. The binding simulations consist of 7 steps in which the equilibrium distance (*r*^N^) was modulated, along with the Gaussian parameters of amplitude and decay *A* and *w*, respectively (see Supplementary Methods). The equilibrium distance for the potential was systematically decreased to allow the relative orientation between the monomers as they approximate towards each other. The Gaussian decay was modified to improve the conformation exploration between molecules. Higher decay values result in wider Gaussian functions and, therefore, in a higher number of possible conformations for a given energy value in the system. Also, the amplitude of the potentials was increased during the last three simulation steps, to further stabilize the complex in the final complex. Each simulation stage was carried until the observation of a stabilized conformation, observed by a reduced variance on each stage’s RMSD. The parameters used for each simulation stage and the variances at the last stage of the simulation are summarized in Supplementary Tables S2-3.

## Additional Information

**How to cite this article**: dos Santos, R. N. *et al.* Dimeric interactions and complex formation using direct coevolutionary couplings. *Sci. Rep.* **5**, 13652; doi: 10.1038/srep13652 (2015).

## Supplementary Material

Supplementary Information

Supplementary Information

## Figures and Tables

**Table 1:** Proteins used to predict dimeric complexes.

Protein	PDB	Length	Family (Pfam)	Sequences
Histidine triad protein	3O0M	149	HIT	8213
GAPDH	4LSM	346	Gp_dh_N	14213
Isocitrate Dehydrogenase	2IV0	412	Iso_dh	12354
Alcohol dehydrogenase	1HDY	374	ADH_zinc_N ADH_N	42002 42970
Aminomutase	2ZSM	434	Aminotran_3	23135
tRNA methyltransferase	1UAL	274	tRNA_m1G_MT	5148
IspG	4G9P	406	GcpE	3640
Ketoacyl synthase	2IX4	431	ketoacyl-synt Ketoacyl-synt_C	24208 23531
Glucose 6-phosphate Isomerase	3FF1	446	PGI	7325
RegX3	2OQR	230	Response_reg Trans_reg_C	47512 47512
PhoB	1ZES	125	Response_reg Trans_reg_C	47512 47512
ATP Corrinoid Adenosyltransferase	1G64	196	CobA_CobO_BtuR	2528
Adenylosuccinate Synthetase	1ADE	431	Adenylsucc_synt	5395
Aspartate Racemase	1JFL	228	Asp_Glu_race	8372
MJ0577 protein	1MJH	162	Usp	22843
3,4-Dihydroxy-2-Butanone 4-Phosphate Synthase	2RIS	204	DHBP_synthase	4699
Zucchini endoribonuclease	4GEL 4GEN	155	PLDc_2	86127
Inositol monophosphatase	2HHM 2QFL	260	Inositol_P	43154

Most proteins are single domain proteins, however for the case of alcohol dehydrogenase and ketoacyl synthase, also interdomain contacts were predicted using two Pfam families. For the case of the response regulator proteins, the additional constrain of being linked to the Trans_reg_C effector domain was used to narrow the number of sequences to a specific subfamily including this regulatory domain.
