# Supplementary material for: Dimeric interactions and complex formation using direct coevolutionary couplings
Source: Sci Rep. 2015 Sep 4;5:13652. doi: 10.1038/srep13652 (PMC4559900; doi:10.1038/srep13652)
Supplement: Supplementary Information [file srep13652-s1.pdf]

Supplementary Information

Dimeric interactions and complex formation using direct coevolutionary couplings

Ricardo N. dos Santos[a,b], Faruck Morcos[a], Biman Jana[c], Adriano D. Andricopulo[b], José N. Onuchic[a]

[a] Center for Theoretical Biological Physics, Rice University, Houston, TX 77005-1827

[b] Laboratório de Química Medicinal e Computacional, Instituto de Física de São Carlos, Universidade de São Paulo, São Carlos, Brazil.

[c] Department of Physical Chemistry, Indian Association for the Cultivation of Science, Jadavpur, Kolkata-700032, India

Supplementary Methods

Structure Based Models with Gaussian Contacts

Structure-Based Models (SBM) have been developed to study folding and conformational changes of proteins through molecular dynamics (MD) simulations[1,2,3]. This approach differs from traditional MD because the potentials (or Hamiltonians) that drive interatomic forces through simulation are parameterized based in a specific reference molecule conformation (usually an experimentally determined structure). On the other hand, usual MD methods employ extensive fixed *ab initio* potentials that are suitable to the study of most biological systems. SBM are constructed following the principle of minimal frustration, where an energy landscape guides protein conformational changes and contains a minimum energy basin corresponding to the known native structure. One advantage of SBM is that they allow the adjustment of the Hamiltonian to consider known interactions that are not present in the reference structure, such as residue-residue interactions observed by experimental studies.

Furthermore, due to its simplicity, modifications and exploration of conformational changes driven by different interactions in the system is straightforward to implement. Therefore, SBM are suitable to study conformational changes related to folding, functional mechanisms and binding in proteins.

The Hamiltonian of a SBM can be represented as follows:

$$H(r_{ij}) = V_B(r_{ij}) + V_{NB}(r_{ij}) \quad (1)$$

where $V_B(r_{ij})$ and $V_{NB}(r_{ij})$ stand for the potentials between covalently bound and non-covalent atom interactions, respectively. The potential that describes covalent interactions can be further described by potentials accounting for each internal degree of freedom of the molecule, as shown above:

$$V_B(r_{ij}) = \sum_{bond} k_b \left(r_{ij} - r_{ij}^{\mathrm{N}}\right)^2 + \sum_{angles} k_a \left(\theta_{ijk} - \theta_{ijk}^{\mathrm{N}}\right)^2 + \sum_{dihedrals} \sum_{n=1,3} k_d^{(n)} \left[1 - cos\left(n\left(\phi_{ijkl} - \phi_{ijkl}^{\mathrm{N}}\right)\right)\right]$$
(2)

where N corresponds to the native reference state used to generate the Hamiltonian. The first term accounts for the energy changes due to variations in the bond length between two atoms. Similarly, the second and third terms consider the energy changes due to variations of binding angles and dihedrals relative to the equilibrium positions, $\theta_{ijk}^{\mathrm{N}}$ and Φ_{ijkl}^{N}, respectively. The constants k_a, k_b and k_d penalize deviations of bonds

and angles from the native structure, biasing the system changes towards the native conformation.

The potential that describes the non-covalent interactions in a SBM Hamiltonian can be expressed as follows:

$$V_{NB}(r_{ij}) = \sum_i \sum_{j>i+3} (1 - C_{ij}) V_{ij}^R(r_{ij}) + C_{ij} V_{ij}^C(r_{ij}) \quad (3)$$

The type of non-bonded interactions can be selected by considering value of $C_{ij} = 1$ for pairs in contact in the contact map of a reference structure and $C_{ij} = 0$ for pairs that do not make contact. The last condition defines $V_{NB}(r_{ij})$ as a simple repulsion interaction that accounts for excluded volume of atoms. The repulsion term in the equation above is defined as:

$$V_{ij}^R(r_{ij}) = \varepsilon \left(\frac{d}{r_{ij}}\right)^{12} \quad (4)$$

where ε is a reduced unit of energy ($\varepsilon = k_b T$) and d is the atomic radius. Historically, SBM Hamiltonian employs a Lennard-Jones (LJ) type potential to stabilize interactions between atoms that are in contact in the reference structure. In the case of coarse grained (C_α) models, this potential is expressed as shown below:

$$V_{ij}^{C}(r_{ij}) = V_{ij}^{LJ}(r_{ij}) = 5\varepsilon\left(\frac{r_{ij}^{\mathrm{N}}}{r_{ij}}\right)^{12} - 6\varepsilon\left(\frac{r_{ij}^{\mathrm{N}}}{r_{ij}}\right)^{10} \quad (5)$$

The shape of this LJ potential is completely defined by the distance between the bead pair r_{ij}. A recently developed and powerful method used in this work allows controlling the form of the non-covalent interaction potential replacing the LJ potential by a similar Gaussian potential that can have its parameters independently modified[4]. This Gaussian potential is defined as follows:

$$V_{ij}^{C}(r_{ij}) = V_{ij}^{G}(r_{ij}) = V_{ij}^{R}(r_{ij}) + A_{ij}G_{ij}(r_{ij}) + V_{ij}^{R}(r_{ij})G_{ij}(r_{ij}) \quad (6)$$

where $G_{ij}(r_{ij})$ is given by:

$$G_{ij}\text{–}r_{ij} = -\varepsilon \exp\left(-\frac{\left(r_{ij}-r_{ij}^{\mathrm{N}}\right)^{2}}{\left(2w_{ij}^{2}\right)}\right) \quad (7)$$

The product $V^{R}_{ij}(r_{ij})G_{ij}(r_{ij})$ ensures that the minimum of the combined potential is at the native distance. The shape parameters A_{ij} and w_{ij} control the amplitude (well depth, or the interaction force factor) and the decay (well width) of the Gaussian SBM potential, respectively.

Supplementary Figures

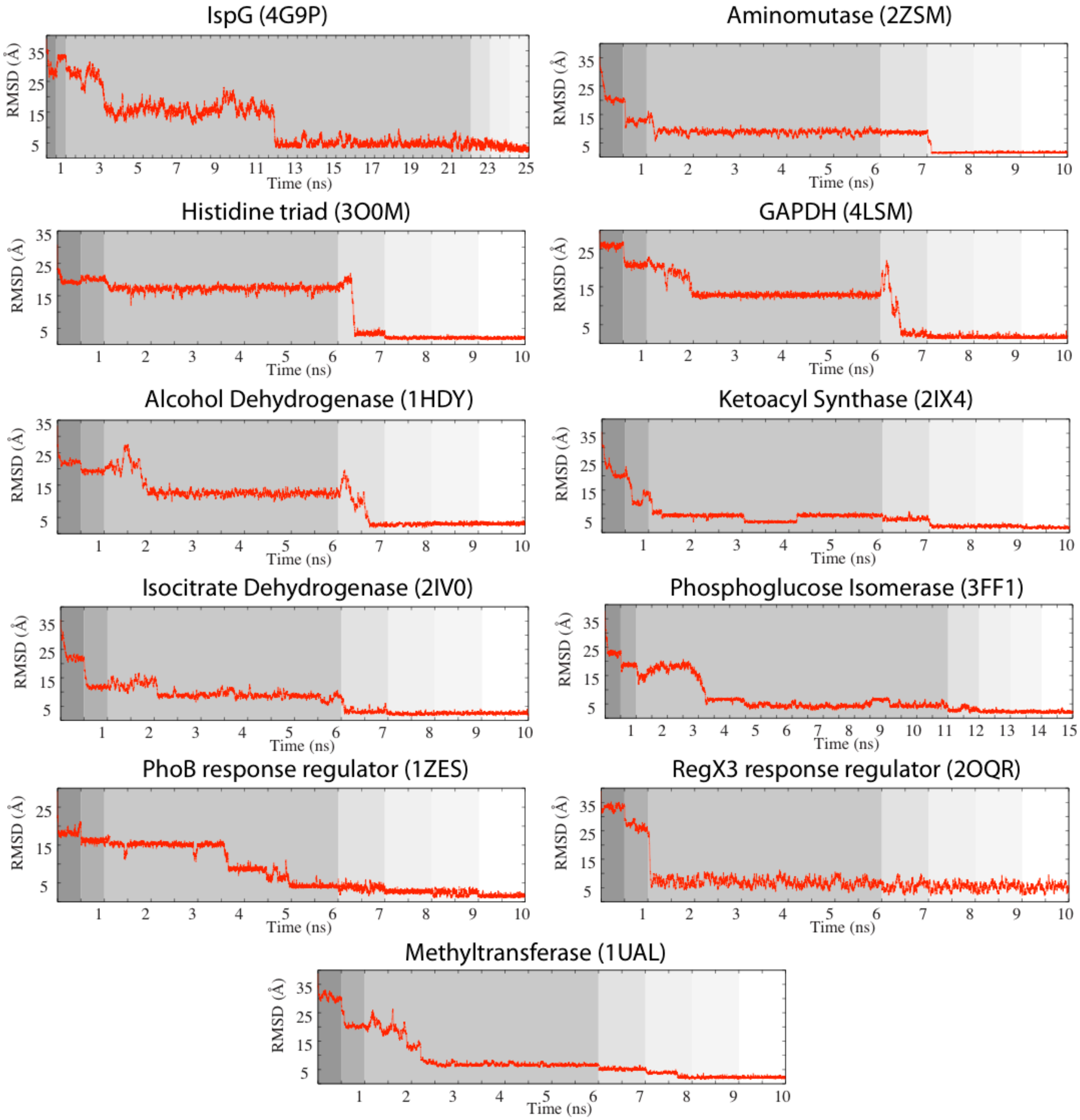

Supplementary Figure S1. RMSD progression of the SBM+DCA methodology for all the proteins studied. The different gray tones indicate different stages where the contact distance parameters are gradually being decreased from 50 Å to 8 Å and the decay *w* of the Gaussian curves is also reduced from 4 to 0.5. The RMSD is computed using the dimeric structures with the PDB accession code shown in each graph. The iRMSD curves have a very similar behavior with slightly lower values in general.

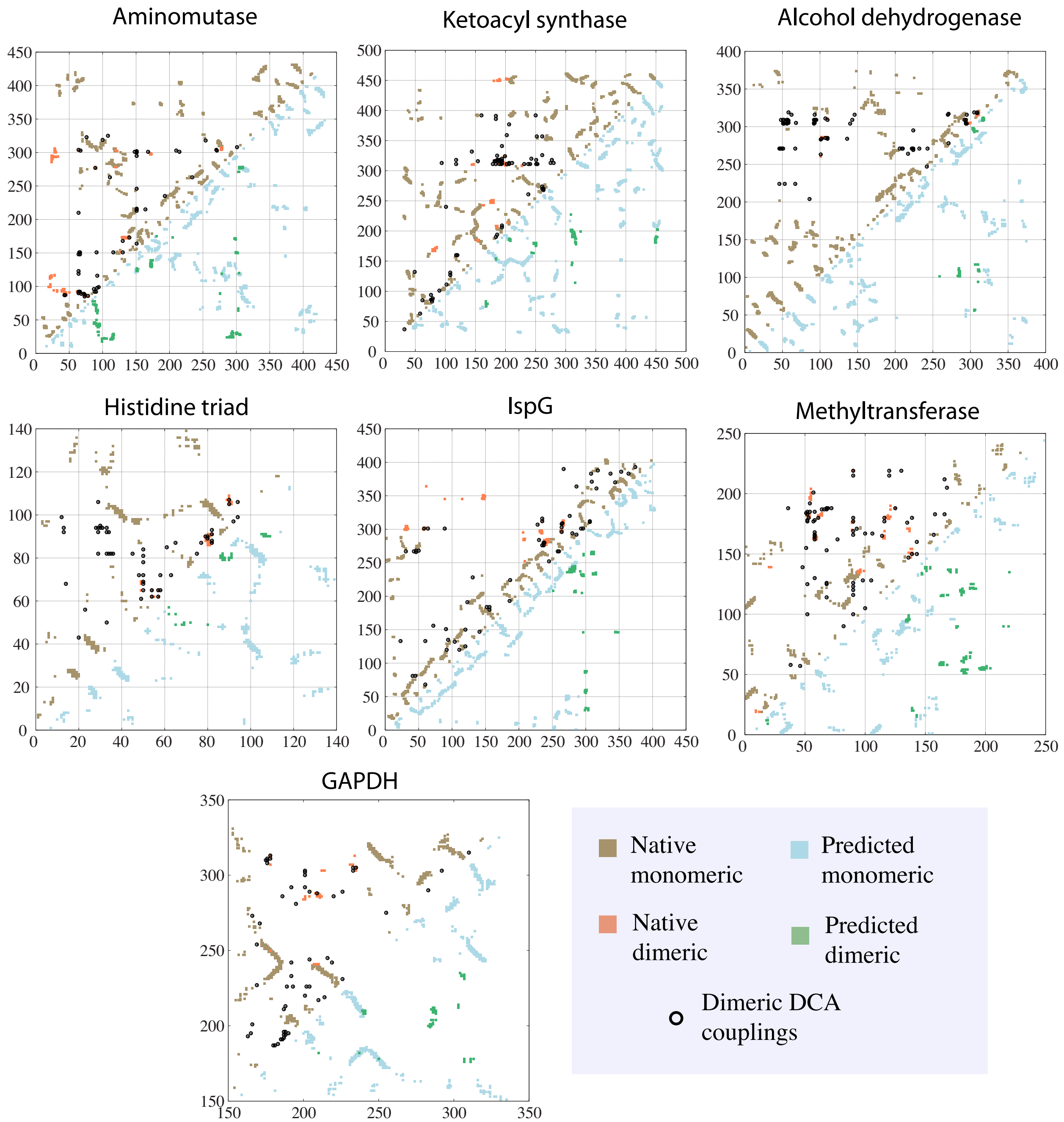

Supplementary Figure S2. Contact maps of different dimeric systems and their predicted contact maps. The upper triangular map shows the native monomeric contacts (brown) along with the native dimeric contacts (orange). The circular symbols represent the top couplings estimated using DCA, the solvent accessibility criterion and removing contacts close to the monomeric map. The lower triangular map shows the best complex prediction. Monomeric contacts are shown in blue and resulting dimeric contacts in green. A comparison between the native dimeric (orange) and predicted dimeric (green) as well as the DCA couplings shows that only a few coevolutionary contacts are needed to be able to recapitulate the remaining contacts that seem to be formed as a consequence of bringing the couplings together.

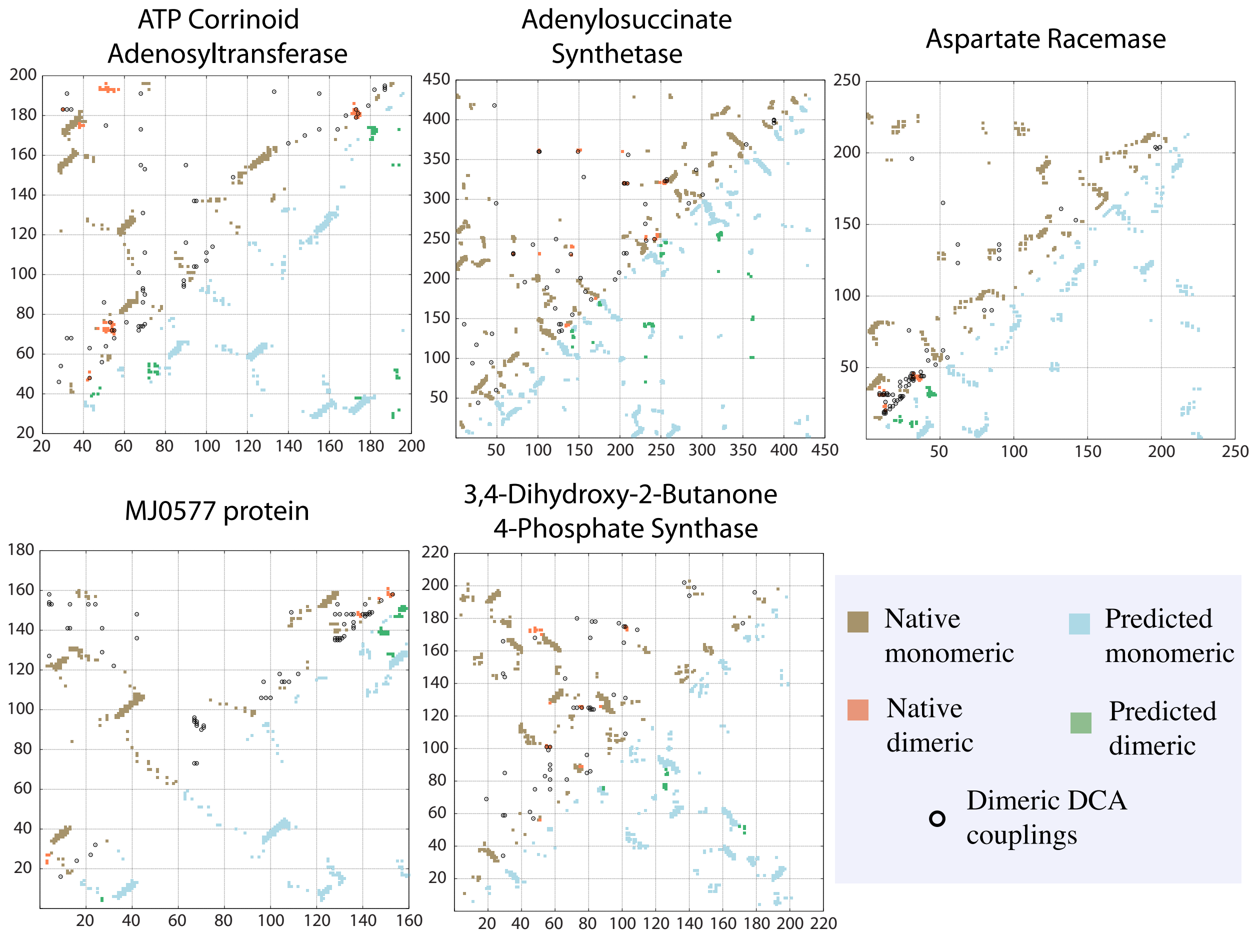

Supplementary Figure S3. Contact maps of different dimeric systems and their predicted contact maps (continued). The upper triangular map shows the native monomeric contacts (brown) along with the native dimeric contacts (orange). The circular symbols represent the top couplings estimated using DCA, the solvent accessibility criterion and removing contacts close to the monomeric map. The lower triangular map shows the best complex prediction. Monomeric contacts are shown in blue and resulting dimeric contacts in green.

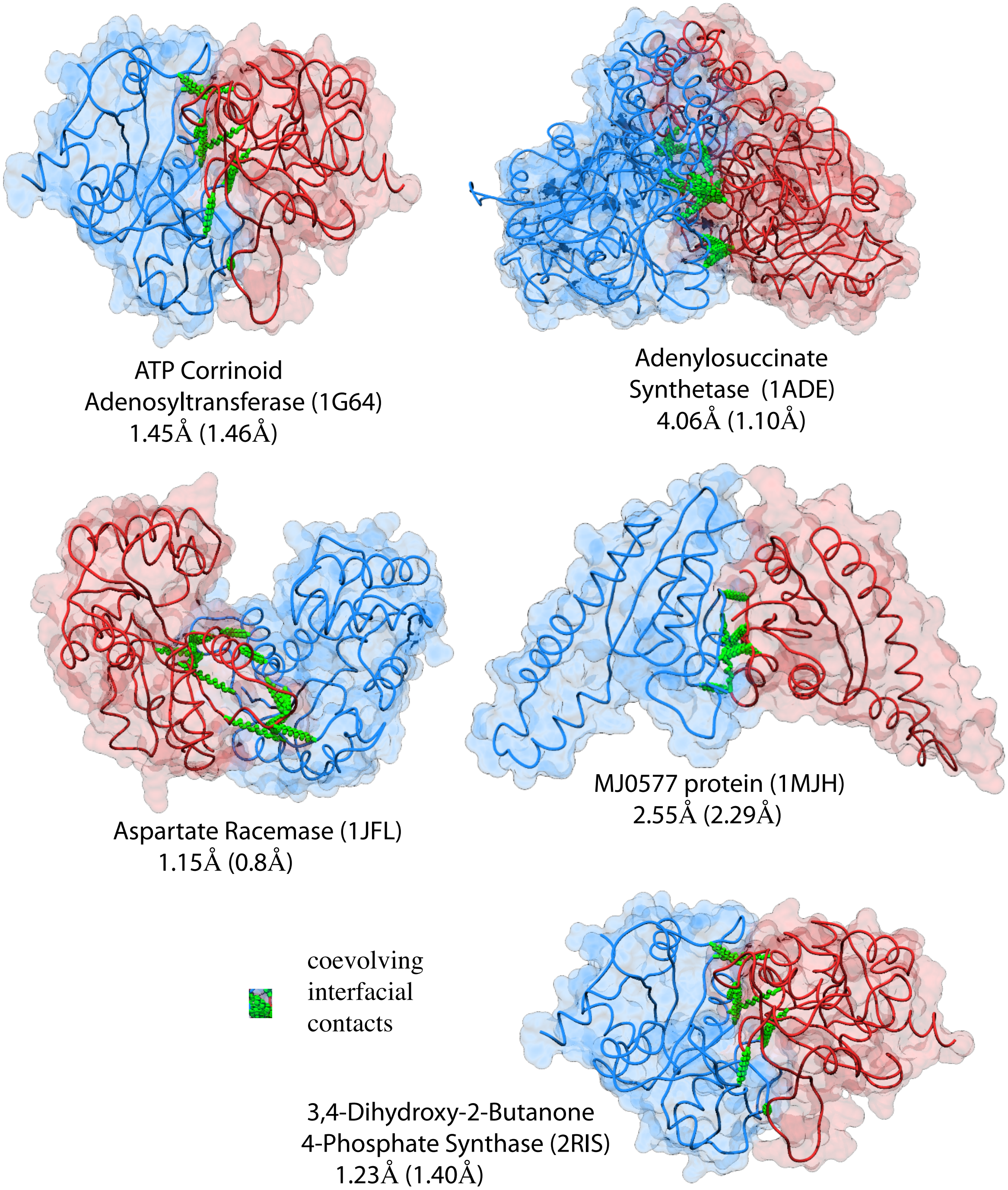

Supplementary Figure S4. Estimated dimeric complexes for 5 additional families. These 5 complexes represent families of medium to large proteins (162-431aa). The iRMSD is particularly low for these cases with an average of 1.41 Å.

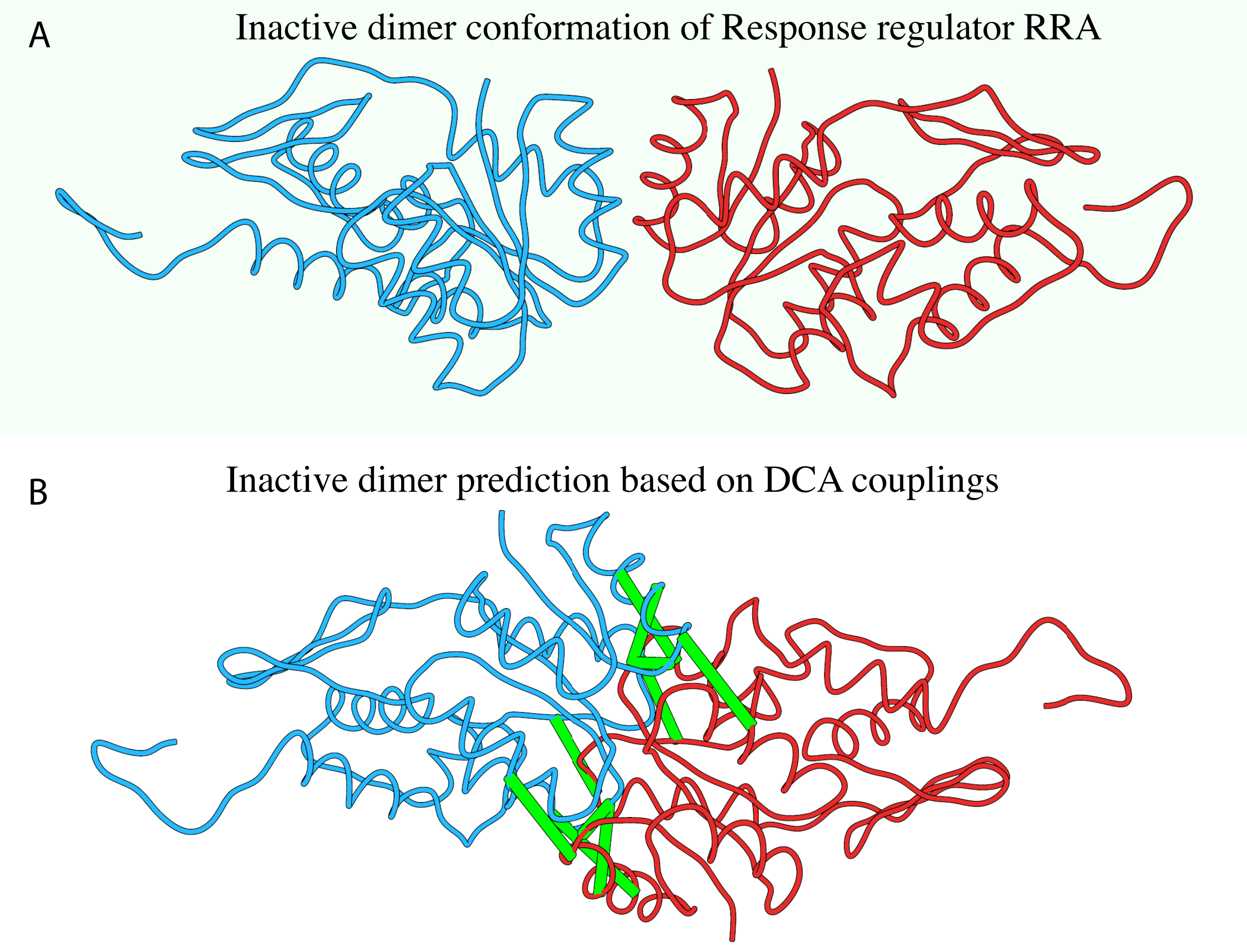

Supplementary Figure S5. The dimeric interface of the suggested inactive state of response regulator RRA is not captured by coevolutionary analysis. (A) Using the monomeric structure of response regulator RRA (PDB 3Q9S) and the suggested inactive interface of only the REC domain in PDB 1B00 we constructed an estimate of the inactive state of the complete response regulator including both receiver and effector domain. (B) Using our methodology we estimated a complex that does not resemble the inactive state and includes signals found in the active state. These results suggest that there is not enough evolutionary signal to support the existence of a physiologically relevant inactive state dimer.

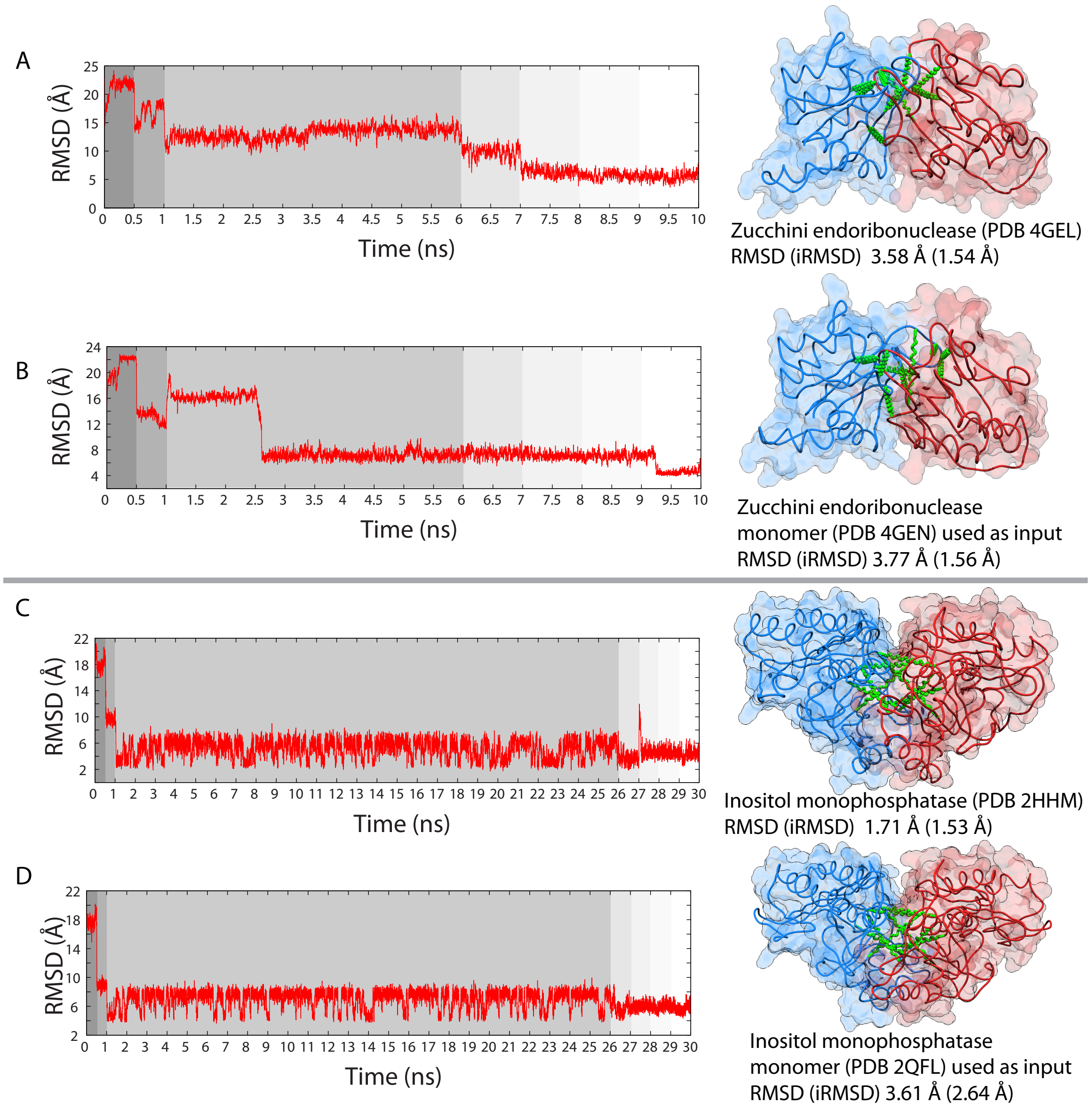

Supplementary Figure S6. The effect of using monomeric vs. dimeric structures as input in our methodology. (A) The dimer of the zucchini endoribonuclease using one structure of its dimer (PDB 4GEL) as input of the simulation. (B) An estimated dimer of the dimer 4GEL using the monomeric structure in the PDB 4GEN. For this case, the outcome of the simulation is very similar for both input structures, especially at the interface. (C) Complex estimated for the dimer of Inositol monophosphatase (PDB 2HHM). (D) The complex of Inositol monophosphatase estimated using monomeric structures as input. There is a larger difference in resolution due to the larger difference in RMSD (3.36 Å) between the monomer and dimeric inputs. See Table S4 for all the comparisons.

Supplementary Tables

Supplementary Table S1. Predictive performance of the SBM+DCA methodology for dimers studied in this work. The comparison with native structures is done using the complex RMSD and iRMSD. The average RMSD/iRMSD was computed using the frames of last stage of the methodology including 2000 structures in each system.

Protein	PDB	Best RMSD	Best iRMSD	Average RMSD	Average iRMSD
Histidine triad protein	3O0M	1.21	1.09	1.98	1.95
GAPDH	4LSM	1.04	0.9	1.71	1.37
Isocitrate Dehydrogenase	2IV0	1.46	1.31	2.57	2.57
Alcohol dehydrogenase	1HDY	1.75	1.56	3.13	2.81
Aminomutase	2ZSM	1.12	1.15	1.67	1.87
tRNA methyltransferase	1UAL	1.50	1.28	2.27	1.80
IspG	4G9P	1.69	1.61	3.15	2.69
Ketoacyl synthase	2IX4	1.09	1.21	1.82	2.10
Glucose 6-phosphate Isomerase	3FF1	1.43	1.468	2.22	2.29
RegX3	2OQR	2.01	1.33	5.085	2.32
PhoB	1ZES	0.89	0.84	1.57	1.67
ATP Corrinoid Adenosyltransferase	1G64	1.45	1.46	2.18	2.23
Adenylosuccinate Synthetase	1ADE	4.06	1.10	4.80	1.69
Aspartate Racemase	1JFL	1.15	0.80	3.80	2.04
MJ0577 protein	1MJH	2.55	2.29	5.12	4.14
3,4-Dihydroxy-2-Butanone 4-Phosphate Synthase	2RIS	1.23	1.40	1.79	1.94

Supplementary Table S2. Parameters of the Hamiltonian used for SBM+DCA simulations.

MD step	parameters		
	r^N (Å)	A	w
1	50	3	2
2	30	3	2
3	15	3	4
4	11	3	4
5	8	5	4
6	8	5	2
7	8	5	0.5

Supplementary Table S3. Standard deviations (Å) of the 16 estimated homo-dimeric structures. These fluctuations or uncertainty in the estimation of the method is computed at the last stage of the simulation protocol. The mean deviation for both RMSD and iRMSD is less than 1 Å.

Protein	PDB	RMSD SD (Å)	iRMSD SD (Å)
Histidine triad protein	3O0M	0.69	0.67
GAPDH	4LSM	0.77	0.67
Isocitrate Dehydrogenase	2IV0	0.82	0.78
Alcohol dehydrogenase	1HDY	0.75	0.64
Aminomutase	2ZSM	0.77	0.79
tRNA methyltransferase	1UAL	0.87	0.80
IspG	4G9P	1.04	0.86
Ketoacyl synthase	2IX4	0.81	0.82
Glucose 6-phosphate Isomerase	3FF1	0.87	0.90
ATP Corrinoid Adenosyltransferase	1G64	0.67	0.68
Adenylosuccinate Synthetase	1ADE	0.76	0.77
Aspartate Racemase	1JFL	1.54	1.12
3,4-Dihydroxy-2-Butanone 4-Phosphate Synthase	2RIS	0.68	0.67
MJ0577 protein	1MJH	0.99	0.70
RegX3	2OQR	0.69	0.29
PhoB	1ZES	0.69	0.70
Zucchini endoribonuclease	4GEL<br>4GEN	0.73<br>1.21	0.48<br>0.40
Inositol monophosphatase	2HHM<br>2QFL	0.72<br>0.57	0.81<br>0.40

Supplementary Table S4. Comparison of the predictive performance for systems with both dimeric and monomeric systems.

Protein	PDB	RMSD between Structures (Cα)	Best RMSD	Best iRMSD	Average RMSD	Average iRMSD
PhoB	1B00 (monomer)	2.10	2.42	2.29	3.77	3.09
	1ZES (ref. dimer)		0.89	0.84	1.57	1.67
Zucchini endoribonuclease	4GEN (monomer)	2.62	3.77	1.56	5.20	3.55
	4GEL (ref. dimer)		3.58	1.54	5.49	3.28
Inositol Monophosphatase	2QFL (monomer)	3.36	3.61	2.64	5.76	4.19
	2HHM (ref. dimer)		1.71	1.53	4.34	4.52

Supplementary Table S5. Diversity of folds in the protein dimeric systems predicted in this study.

Protein	PDB	SCOP fold classification
Histidine triad protein	3O0M	HIT-like
GAPDH	4LSM	NAD(P)-binding Rossmann-fold domains
Isocitrate Dehydrogenase	2IV0	Isocitrate/Isopropylmalate dehydrogenase-like
Alcohol dehydrogenase	1HDY	GroES-like
Aminomutase	2ZSM	PLP-dependent transferase-like
tRNA methyltransferase	1UAL	alpha/beta knot
IspG	4G9P	Terpenoid synthases*
Ketoacyl synthase	2IX4	Thiolase-like
Glucose 6-phosphate Isomerase	3FF1	Double-stranded beta-helix
RegX3	2OQR	DNA/RNA-binding 3-helical bundle<br>Flavodoxin-like
PhoB	1ZES<br>1B00	Flavodoxin-like
ATP Corrinoid Adenosyltransferase	1G64	P-loop containing nucleoside triphosphate hydrolases
Adenylosuccinate Synthetase	1ADE	P-loop containing nucleoside triphosphate hydrolases
Aspartate Racemase	1JFL	ATC-like
MJ0577 protein	1MJH	Adenine nucleotide alpha hydrolase-like
3,4-Dihydroxy-2-Butanone 4-Phosphate Synthase	2RIS	YrdC/RibB
Zucchini endoribonuclease	4GEL<br>4GEN	Cupredoxin-like
Inositol Monophosphatase	2HHM<br>2QFL	Carbohydrate phosphatase

Supplementary Movies

Supplementary Movie S1. The movie shows the output frames of a simulation using the SBM+DCA protocol for protein tRNA methyltransferase. At the beginning the monomers are unbound but as simulation progresses dimer formation occurs until reaching a RMSD of 1.5 Å with respect to the experimental structure PDB 1UAL.

Supplementary References

1. Jana, B., Morcos, F. & Onuchic, J. N. From structure to function: the convergence of structure based models and co-evolutionary information. *Physical chemistry chemical physics : PCCP* **16**, 6496-6507, (2014).
2. Onuchic, J. N. & Wolynes, P. G. Theory of protein folding. *Current Opinion in Structural Biology* **14**, 70-75, (2004).
3. Whitford, P. C., Sanbonmatsu, K. Y. & Onuchic, J. N. Biomolecular dynamics: order-disorder transitions and energy landscapes. *Rep Prog Phys* **75**, (2012).
4. Lammert, H., Schug, A. & Onuchic, J. N. Robustness and generalization of structure-based models for protein folding and function. *Proteins Struct Func Bioinf* **77**, 881-891, (2009).
